# The Effects of Yoghurt Acid Whey Marination on Quality Parameters of Pork and Chicken Meat

**DOI:** 10.3390/foods12122360

**Published:** 2023-06-13

**Authors:** Agori Karageorgou, Anastasia Paveli, Michael Goliomytis, Georgios Theodorou, Ioannis Politis, Panagiotis Simitzis

**Affiliations:** Laboratory of Animal Breeding and Husbandry, Department of Animal Science, Agricultural University of Athens, 75 Iera Odos, 11855 Athens, Greece; akarageorgou@aua.gr (A.K.); mgolio@aua.gr (M.G.); gtheod@aua.gr (G.T.); i.politis@aua.gr (I.P.)

**Keywords:** yoghurt acid whey, chicken meat, pig meat, shear force, color parameters, oxidative stability

## Abstract

Large amounts of yoghurt acid whey (YAW) are annually generated as a result of Greek-style yoghurt production, which poses a great hazard to the environment. In terms of sustainability, YAW application in the meat industry appears as a great alternative since meat marination with natural solutions is a practice that continuously gains ground due to its positive effects on meat sensory attributes. The aim of the present study was to determine the quality characteristics and oxidative status of pork and chicken meat after their marination in yoghurt acid whey. Forty samples per meat type were randomly assigned into five groups: CON, without YAW marination; YAW1 and YAW3, in which meat was marinated at 4 °C and a pH of 4.5 for 15 and 10 h, respectively; or YAW2 and YAW4, in which meat was handled as in the YAW1 and YAW3 group, respectively, while hesperidin at 2 g/L was also incorporated into the marinade. As shown, meat shear force values were decreased in pork but not in chicken meat samples. Meat pH values were also generally decreased, while lightness was increased in raw but not in cooked meat samples as an effect of marination. Moreover, meat oxidative stability was improved to a greater extent in chicken than in pork meat. In order to find the ideal marination period for pork meat, we further immersed it into YAW for 5 h. However, this treatment affected neither meat tenderness and the other quality properties nor meat oxidation rates. In general, hesperidin addition did not have an additional or side effect on the quality traits of pork and chicken meat. As it can be concluded, pork meat marination in YAW for 10–15 h improves tenderness, but 5 h of marination does not. On the other hand, the tenderness of chicken meat was not affected, but its oxidative stability was greatly improved after remaining in the YAW marinade for 10–15 h.

## 1. Introduction

According to the FAO [1], yoghurt production is constantly increasing in Greece, reaching 195,510 tn in 2020. Consequently, the large amounts of yoghurt acid whey (YAW) obtained increase the risk of environmental pollution since YAW is regarded as a strong pollutant because of its high organic matter and biological oxygen demand (BOD) [2]. YAW is the by-product of acid coagulation, which is accomplished by fermentation using lactobacilli or the addition of organic or mineral acids [3]. YAW is a dairy by-product for which the industry has long struggled to find a sustainable utilization [4]. The most common application of YAW is on farmland as a fertilizer that is either directly added to the soil or mixed with manure before its application [5]. Another common use for acid whey is as an ingredient in animal diets since it can be mixed with silage and fed to livestock [6]. Finally, the lactose contained in YAW could be used as a substrate to produce valuable compounds via fermentation and to generate energy in wastewater bioreactors [7,8].

In recent years, environmentally conscious dairy food companies have been working to develop a sustainable solution for YAW utilization via its careful valorization and commercialization. At the same time, the recent advancements in whey processing technologies, which include ultrafiltration, microfiltration, and diafiltration membrane and ion-exchange technologies, currently provide high-quality whey fractions for product and application development. These fractions are characterized by high nutritional quality as well as intense antimicrobial and antioxidant properties [9]. As a result, their use is extended in diverse food systems such as binders, egg white substitute, or flavor enhancers [10]. Especially in the meat industry, the addition of acid whey in sausages [11] and dry-cured pork loins [12] was shown to provide a protection against oxidative deterioration without negatively influencing their physicochemical characteristics and sensory attributes. Moreover, naturally derived agents such as citrus by-products have already been added to marinades to further improve meat oxidation stability [13]. Hesperidin is a flavonoid contained in citrus fiber and is well known for its antioxidant properties [14].

A tendency observed in the global market of meat products is the growing demand for “convenience” products, in which marinated products are among the most desired [15]. The use of marinades using natural additives may determine the desired taste and flavor profile of the product and induce beneficial effects on the consumer’s health, as well as the originality of the dish [16]. Although YAW has a lower protein content, it has higher levels of calcium and lactic acid and, as a result, a lower pH value compared to the sweet whey derived from cheese [2,7]. Lactic acid abundance makes YAW an ideal candidate as a tenderizer agent since marination in acidic solutions has been traditionally used as a means of softening and flavoring meats and decreasing their mechanical resistance [17]. A preliminary study showed that YAW marination for 20 h could be suggested as an innovative procedure that improves pork, sheep, and rabbit meat tenderness, while the other quality characteristics assessed just after marination were not negatively affected [18]. Tenderness is one of the major organoleptic characteristics that the consumer associates with meat palatability and is therefore crucial for their choice. Owing to this fact, the meat industry is continuously struggling to discover innovative strategies that could provide softer meat products, and one promising process of achieving this target is acid whey marination [17]. In a recent study, the marination of hen breast meat with buttermilk and acid whey for 24 and 48 h [19] or with buttermilk and sour milk for 12 h [20] resulted in a greater value for lightness, while shear force, hardness, and chewiness decreased.

Results referring to the effects of yoghurt acid whey marination on pork and chicken meat quality traits are insufficient. In our previous preliminary study [18], meat samples were immersed into YAW for 20 h, and their quality characteristics were evaluated only in raw and cooked meat samples on day 1. Our hypothesis is that the use of YAW as a marinade could provide a dual benefit of improving tenderness while also inhibiting lipid oxidation even after shorter periods of immersion. As a consequence, the aim of the present study was to point out the potential amelioration in quality parameters and oxidative stability of pork and chicken meat as an effect of YAW marination for 5–15 h and after 1–9 or 30–60 days of refrigerated and frozen storage, respectively. 

## 2. Materials and Methods

### 2.1. Samples and Marination Procedure

Pork and chicken meat samples were collected from 6-month-old pigs and 42-day-old broilers slaughtered in a commercial abattoir to ensure similar animal husbandry and slaughtering conditions and were transferred to the laboratory on ice and stored at a refrigerator (4 °C) until their analysis, which was carried out within 5 h. In brief, we have dissected *longissimus thoracis* muscle between the 6th and 13th rib for pork and *pectoralis major* muscle for chicken meat quality assessment analyses. Meat samples originated from eight pigs or broilers, and each sample was separated into five sub-samples that were randomly assigned to either the control group (C1), which was not immersed in YAW, or one of the following four marination groups: YAW1 or YAW3, in which the samples were immersed into YAW for 15 and 10 h, respectively, at 4 °C; or YAW2 or YAW4, in which they were kept in an acid whey marinade for 15 and 10 h, respectively, at 4 °C with the simultaneous inclusion of hesperidin (TSI Europe NV, Zwijndrecht, Belgium) at the concentration of 2 g/L based on our preliminary study [18]. Hesperidin is a flavanone glycoside of citrus fruits with multifunctional biological properties, which is especially known for its antioxidant activity [21]. For pork meat, a second experiment was also carried out to find its ideal marination period. In this case, pork meat samples originated from eight pigs, and each sample was further divided into four sub-samples randomly assigned to four groups: control (C2), which was not immersed in YAW; YAW5, in which the samples remained in acid whey marinade for 5 h at 4 °C; YAW6, in which the samples were immersed into YAW for 5 h at 20 °C; or YAW7, with the same conditions as YAW6 but with a concomitant addition of hesperidin at the level of 2 g/L.

YAW used in the current experiment was derived from cow milk originated from various small-sized dairy farms in Greece. YAW marinade was formulated by mixing yoghurt serum powder with distilled water till the desirable pH value (approximately 10 g YAW per 100 mL for obtaining pH value of 4.5). Yoghurt serum was mechanically derived after the fermentation of authentic Greek-style strained yoghurt with *Streptococcus thermophilus* and *Lactobacillus bulgaricus* in a dry free-flowing powder with an ash and moisture level of 11 and 4%, respectively, which contained 72% lactose, 6% lactic acid, 8.5% galactose, and 5% protein in addition to 24.7 g potassium, 18 g calcium, 14.4 g chloride, 6.6 g sodium, 6 g phosphorus, 1.7 g magnesium, 1.13 mg ferrum, and 0.48 mg copper per kg (Epirus Protein S.A., Ioannina, Greece).

### 2.2. Meat Quality Assessment

#### 2.2.1. Marinade Absorption, Tenderness, and Cooking Loss

Raw meat samples (80 ± 2 g) were weighed before and after their immersion into YAW marinade for the determination of marinade absorption, which was calculated as a percentage of the initial meat sample weight (%). Meat samples were then placed in plastic bags and cooked in a water bath at the following conditions according to meat type: 50 min at 75 °C for pork [22] and 30 min at 80 °C for chicken [23] meat. The samples were subsequently cooled under running water for 15 min and then equilibrated to room temperature. The samples were weighed again in order to estimate cooking loss (%), which is an index of water-holding capacity [24]. Three sub-samples with a cross section of 1 cm^2^ were cut parallel to the muscle fibers, and their shear force value was determined using a Warner–Bratzler (WB) shear blade fitted to a Zwick Testing Machine Model Z2.5/TN1S (Zwick GmbH & Co., Ulm, Germany). Peak force values were measured in newtons. Warner–Bratzler shear force evaluation is a classic instrumental method for the estimation of meat tenderness (toughness) [25].

#### 2.2.2. pH and Color Parameters

Acidity (pH) values were assessed by a pH meter (HI 99163 model, Hanna instruments, Romania) with the electrode inserted into raw and cooked meat samples on day 1 and on days 3, 6, and 9 after marination and storage at 4 °C. Buffers of pH 4.0 and 7.0 (Merck, Darmstadt, Germany) were used for the standardization of the pH meter at room temperature (~20 °C). Color parameters were also determined in raw and cooked meat samples on day 1 and on days 3, 6, and 9 after marination and storage at 4 °C, using a Miniscan XE (HunterLab, Reston, VA, USA) chromameter, which was calibrated using a black and white tile and set on the L* (lightness), a* (redness), and b* (yellowness) systems (CIE 1976, Commission International de l’ Eclairage, Vienna, Austria).

#### 2.2.3. Oxidative Stability

Malondialdehyde (MDA) values were determined on day 1; on days 3, 6, and 9 after storage at 4 °C; and on days 30 and 60 after storage at −20 °C by a selective third-order derivative spectrophotometric method as an index of lipid oxidation [26]. In brief, 2 g from each raw or cooked meat sample (two replications) were mixed with 8 mL aqueous trichloroacetic acid (TCA) (50 g/L) and 5 mL butylated hydroxytoluene (BHT) in hexane (8 g/L) and homogenized (Unidrive × 1000, CAT, M. Zipperer GmbH, Ballrechten-Dottingen, Germany). The obtained mixture was centrifuged for 5 min at 5000× *g*. After the disposal of hexane, a 2.5 mL aliquot from the bottom layer was mixed with 1.5 mL aqueous 2-thiobarbituric acid (TBA) (8 g/L), and the mixture was further incubated at 70 °C for 30 min. The mixture was then left under tap water to cool, and third-order derivative (3D) spectrophotometry (Hitachi U3010 Spectrophotometer) in the range of 500–550 nm was applied. Meat MDA levels (ng/g wet tissue) were calculated on the basis of the height of the third-order derivative peak at 521.5 nm by contrasting the intercept data with the slope of the standard calibration curve prepared using the MDA precursor 1,1,3,3-tetraethoxypropane (TEP).

### 2.3. Statistical Analysis

Marinade absorption, cooking loss, and shear force value measurements were analyzed using a mixed-model procedure, which contained the fixed effect of marination treatment. Data referring to pH values, color parameters, and MDA concentration were analyzed using a mixed-model procedure appropriate for repeated measurements per subject, which included marination treatment as fixed effect. Mean differences were detected at a 0.05 significance level with Bonferroni adjustment. Analyses were conducted by Sas/Stat [27]. Results are presented as least squares (LS) means ± standard error of mean (S.E.M.).

## 3. Results

### 3.1. Pork Meat

**Figure 1:**
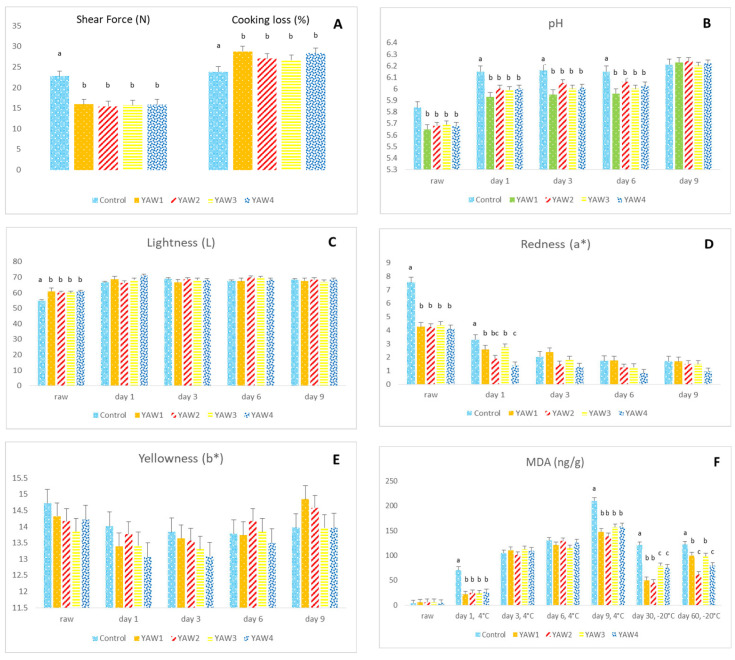
Effect of YAW marination of pork meat for 10–15 h on (**A**) shear force (N) and cooking loss (%); (**B**) pH; (**C**) lightness; (**D**) redness; (**E**) yellowness; and (**F**) MDA (ng/g). The treatment groups included control (C1), without marination, or one of the four marination treatments, including YAW1 and YAW3, in which the samples remained in YAW for 15 and 10 h, respectively, at 4 °C, or YAW2 and YAW4, in which the samples remained in YAW for 15 and 10 h, respectively, at 4 °C with a concomitant addition of hesperidin at the level of 2 g/L. ^a, b, c^ Values with different superscripts are significantly different.

The values for pork meat marinade absorption (%) were not significantly different among the YAW groups (5.44 ± 0.33 vs. 5.25 ± 0.33 vs. 5.43 ± 0.33 vs. 5.31 ± 0.33 for YAW1, YAW2, YAW3, and YAW4 groups, respectively; *p* > 0.05). As illustrated in Figure 1, the pork meat shear force values were reduced, and, consequently, tenderness was improved after immersion into YAW (Figure 1A; *p* < 0.001). On the other hand, cooking loss (%) was increased as a result of pork meat immersion into YAW (Figure 1A; *p* < 0.05). Moreover, meat immersion into YAW resulted in a reduction in pH values in both raw and cooked samples from day 1 till day 6 after refrigerated storage (Figure 1B; *p* < 0.05). Lightness (L*) values were enhanced in the raw meat samples that were marinated with YAW (*p* < 0.05), but this impact was diminished in cooked samples, and, as a result, no significant differences were observed among the experimental groups (*p* > 0.05) (Figure 1C). On the other hand, redness (a*) values were reduced as an effect of marination in both raw and cooked meat samples on day 1 (*p* < 0.05), whereas no significant differences were observed among the groups on days 3, 6, and 9 (*p* > 0.05) (Figure 1D). Yellowness (b*) values were not affected by YAW marination (Figure 1E; *p* > 0.05). Finally, YAW marination significantly influenced the oxidative stability of pork meat since the MDA values were decreased on days 1 and 9 at 4 °C and on days 30 and 60 at −20 °C (Figure 1F; *p* < 0.01). The addition of hesperidin in the YAW marinade further improved pork meat oxidative stability after 60 days of frozen storage, as shown from the reduced MDA values of the YAW2 and YAW4 group samples in comparison with the YAW1 and YAW3 group samples (Figure 1F; *p* < 0.05).

**Figure 2:**
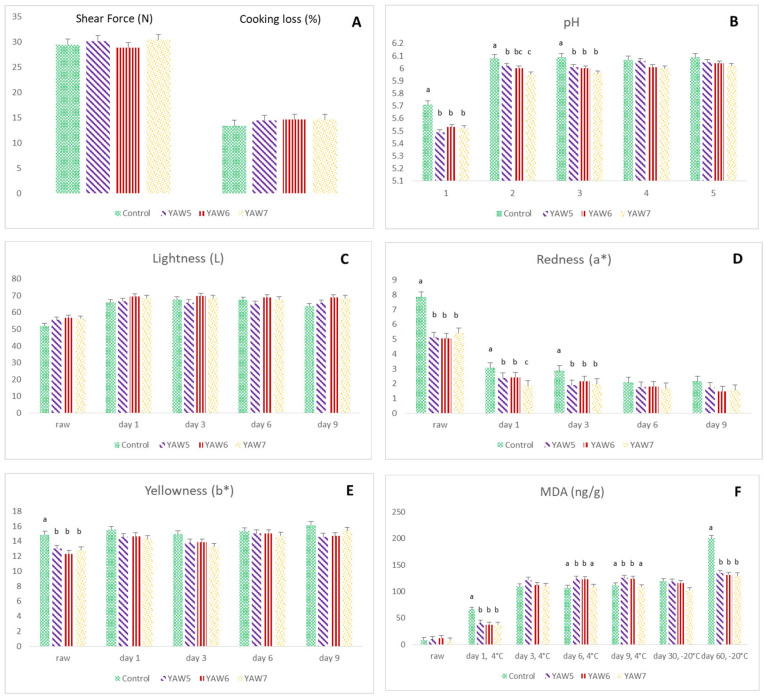
Effect of YAW marination of pork meat for 5 h on (**A**) shear force (N) and cooking loss (%); (**B**) pH; (**C**) lightness; (**D**) redness; (**E**) yellowness; and (**F**) MDA (ng/g). The treatment groups included control (C2), without marination; YAW5, in which the samples remained in acid whey marinade for 5 h at 4 °C; YAW6, in which the samples remained in acid whey marinade for 5 h at 20 °C; and YAW7, with the same conditions as YAW6 but with a concomitant addition of hesperidin at the level of 2 g/L. ^a, b, c^ Values with different superscripts are significantly different.

As previously mentioned, in an effort to evaluate the efficacy of YAW as a pork meat tenderizer, a further experiment was carried out in which meat remained in acid whey marinade for 5 h. The values for pork meat marinade absorption (%) were also not significantly different among the YAW groups (3.93 ± 0.09 vs. 3.94 ± 0.09 vs. 3.85 ± 0.09 for YAW5, YAW6, and YAW7 group, respectively; *p* > 0.05). However, these values were lower compared to those after YAW marination for 10–15 h. As shown in Figure 2, the results were not promising, like in the immersion for 10–15 h, since the shear force values were not affected by the immersion into YAW for 5 h (Figure 2A). Cooking loss (Figure 2A) and lightness (Figure 2C) were also not affected (*p* > 0.05). pH (Figure 2B) and redness (a*) (Figure 2D) showed a reduction in their values in both raw and cooked samples from day 1 till day 3 after refrigerated storage as a result of YAW marination (*p* < 0.05). Yellowness (b*) only showed a decrease in raw pork meat samples in the YAW groups (Figure 2E; *p* < 0.05). Nevertheless, YAW marination for 5 h significantly improved the oxidative stability of pork meat since the MDA values decreased on day 1 at 4 °C and on day 60 at −20 °C (Figure 2F; *p* < 0.01).

### 3.2. Chicken Meat

**Figure 3:**
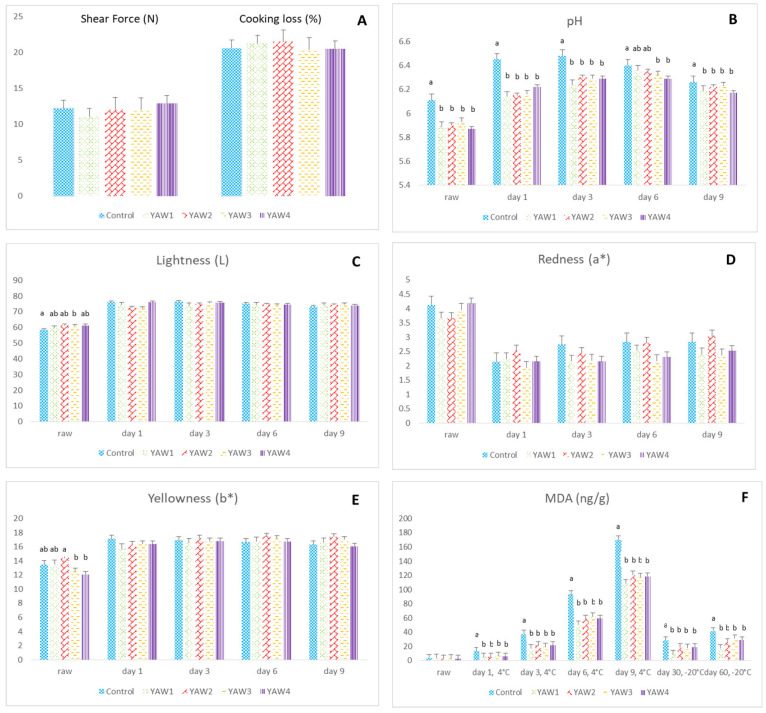
Effect of YAW marination of chicken meat for 10–15 h on (**A**) shear force (N) and cooking loss (%); (**B**) pH; (**C**) lightness; (**D**) redness; (**E**) yellowness; and (**F**) MDA (ng/g). The treatment groups included control (C1), without marination, or one of the four marination treatments, including YAW1 and YAW3, in which the samples remained in YAW for 15 and 10 h, respectively, at 4 °C, or YAW2 and YAW4, in which the samples remained in YAW for 15 and 10 h at 4 °C with a concomitant addition of hesperidin at the level of 2 g/L. ^a, b^ Values with different superscripts are significantly different.

The values for chicken meat marinade absorption (%) were not significantly different among the YAW groups (4.97 ± 0.37 vs. 5.14 ± 0.37 vs. 5.18 ± 0.37 vs. 5.22 ± 0.37 for YAW1, YAW2, YAW3, and YAW4 groups, respectively; *p* > 0.05). As shown in Figure 3, the chicken meat shear force values were not affected by YAW marination, and, consequently, meat tenderness was not improved (Figure 3A; *p* > 0.05). Cooking loss (%) (Figure 3A) and redness (a*) (Figure 3D) were similarly not affected by YAW treatment. On the other hand, YAW marination caused a reduction in meat pH values in both raw and cooked samples from day 1 till 9 after refrigerated storage (Figure 3B; *p* < 0.05). Lightness (L*) values were increased in the raw meat samples that were marinated with YAW, with a significant difference between YAW3 and the control group (*p* < 0.05), but this effect was diminished in the cooked samples (*p* > 0.05) (Figure 3C). The yellowness (b*) value was higher in the YAW2 group compared to the YAW3 and YAW4 groups in the raw samples (*p* < 0.05), but no significant differences were observed in the cooked chicken meat samples (Figure 3E). Finally, YAW marination significantly influenced the oxidative stability of chicken meat since MDA values were decreased on days 1, 3, 6, and 9 at 4 °C and on days 30 and 60 at −20 °C (Figure 3F; *p* < 0.01). The addition of hesperidin in the YAW marinade did not alter meat oxidative stability, as indicated by the lack of any difference between the YAW1 and YAW3 group samples and between the YAW2 and YAW4 group samples (*p* > 0.05).

## 4. Discussion

One of the most widely used techniques to enhance the flavor and improve the tenderness of meat is marination. Soaking or immersion is a traditional method for producing marinated meat products and consists of submerging the meat in the marinade and allowing the ingredients to penetrate the meat via diffusion with the passage of time [28]. As indicated in the current experimentation, the high content of lactic acid and calcium in YAW contributed to decreased values for shear force in pork meat. Similar results were also reported in pork meat immersed in lactic acid water-based mixtures (1–3%) for 1–3 min [29]. On the other hand, the immersion of chicken meat into YAW did not influence tenderness. In contrast, Augustyńska-Prejsnar et al. [30] showed that marination into YAW for 12 h dropped turkey meat shear force values. Ergezer and Gokce [31] also reported a reduction in the shear value of turkey breast meat marinated with lactic acid. The marination of hen breast meat with buttermilk and acid whey for 24 and 48 h also resulted in decreased values for shear force, hardness, and chewiness, indicating an improvement in tenderness [19]. Similar findings were found after marinating hen breast meat in buttermilk and sour milk for 12 h at 4 °C [20]. The tenderness of chicken meat fillets was also improved after their immersion in whey for 12–24 h [32] or 24 h [33]. In our preliminary study, in which meat samples were immersed in yoghurt acid whey for 20 h, tenderness was improved in pork but not in chicken meat samples [18]. In other meat types, no effect of YAW marination for 24 h on beef cutting force values were observed [34], while beef tenderness was improved after treatment with lactic acid [35,36] or calcium salts [37]. Lamb meat tenderness was also improved after its immersion into marinades based on acid whey and other food industry by-products for 24–48 h [38]. The improvement in meat tenderness due to acid marination could be an effect of the loosening of the structure in collagen connective tissue. According to Kumar et al. [39], the acid breaks the transversal bounds of collagen, leading to the unstable structure loss of this connective tissue protein. In brief, acid-labile cross linkages in collagen molecules are released, causing a connective tissue breakdown and, especially, perimysial tissue degradation [17,36]. However, the efficacy of YAW as a tenderizer agent was not the same in pork and chicken meat, and this discrepancy could be attributed to the fact that marination seemed to be more effective in softening muscles with a high content of connective tissue [17].

The immersion of meat into YAW generally resulted in a decrease in meat pH values, revealing significant differences in raw and cooked pork and chicken meat corresponding to the acidity of the YAW marinade. A significant drop in pH values following immersion in YAW for 20 h has been previously reported by our group [18]. Immersion in lactic acid has also been reported to decrease pH values in beef [35] and pork [29] meat. Acid whey marination also caused a decrease in pH values in turkey [30], chicken [33], and pheasant [40] breast meat. Wojciak et al. [41] arrived at similar findings after the immersion of fermented beef in acid whey. In general, acidic conditions, i.e., pH values within 5.2–5.5, are associated with improved tenderness in beef muscle [42]. The effects of marinades are often associated with swelling and the enhanced extraction of myofibrillar proteins and are correlated with a decrease in pH and an increase in ionic strength [43,44].

Lightness was enhanced in raw pork and chicken meat samples marinated for 10–15 h, but this effect was not shown in cooked meat. This is of great significance especially for pre-cooked meat products since the pale color of YAW-treated raw samples is lost due to the cooking procedure, and the cooked samples have similar values for brightness with the controls. These changes were also reported in our previous study, in which meat samples remained in YAW for 20 h [18]. Differences in lightness could be attributed to pH reduction since the proteolysis of sarcoplasmic and myofibrillar proteins by endogenous (calpains and cathepsins) and exogenous (originated from acid whey) endopeptidases may influence their water-binding ability. The greater amount of extracellular water introduced into the meat during marinating and scattered among the muscle fibers could affect meat reflectance ability [36]. In contrast to lightness, redness (a*) decreased in raw and cooked pork meat samples after immersion into YAW for 5–15 h. A similar finding was reported in hen breast meat samples marinated in YAW for 24–48 h [19]. At the same time, a significant decrease in the yellowness (b*) values of raw pork and chicken meat samples was observed after YAW marination for 5 and 10–15 h, respectively. Similar results were shown in our preliminary study with an immersion of pork and chicken meat in YAW for 20 h [18]. In contrast to our findings, pork meat marination in a lactic acid solution did not induce a significant effect on its color [34]. On the contrary, YAW marination resulted in an increase in lightness and yellowness in raw and roasted turkey meat samples, whereas redness was not influenced [30]. In hen breast meat samples, lightness was increased as an effect of marination with buttermilk and whey for 24 and 48 h [19]. The same research group reached similar conclusions after the marination of raw pheasant breast muscles with acid whey for 24 h [40]. On the contrary, the color parameters of beef were not influenced as an effect of YAW marination [34], while increments in redness and yellowness were shown after the immersion of fermented beef eye round in acid whey [41]. An increase in lightness and a decrease in redness were also reported in beef cuts immersed in a lactic acid aqueous mixture, while yellowness was not affected [36,45].

Finally, YAW marination did not hasten the meat oxidation process since MDA values were, in general, decreased as an effect of YAW marination, and this effect was more evident in chicken compared to pork meat samples. This finding is in accordance with our preliminary study, in which marinating meat in YAW for 20 h decreased MDA values on day 1, with significant differences only for chicken meat [18]. The immersion of fermented beef into acid whey also maintained oxidative stability during storage [41]. On the other hand, Wojciak et al. [34] showed enhanced values for 2-thiobarbituric acid reactive substances (TBARS) in beef immersed in acid whey after 1 day and 28 days but a decreased TBARS concentration after 14 days of storage. Furthermore, as pointed out in the current study, the inclusion of hesperidin into the marinade did not have an additional beneficial effect on meat oxidative stability (apart from pork meat after 60 days of frozen storage), although it is well known that hesperidin is a bioactive compound that can actively scavenge free radicals [14]. This discrepancy could be possibly attributed to the fact that YAW already reduced lipid oxidation rates to such an extent that hesperidin could not act synergistically.

There are some limitations in our study that may restrict the conclusions we can draw from its results. For example, no sensory evaluation of cooked pork and chicken was carried out. Unfortunately, our experiment was implemented during the COVID-19 outbreak, and it was difficult to find candidates to form the sensory evaluation team consisting of people with confirmed sensory sensitivity and with experience in the field of sensory evaluation. Moreover, analyses such as the calculation of protein oxidation values, the determination of volatile compounds, texture profile analysis, and the assessment of microbiological parameters could strengthen our understanding of the effects of YAW marination on meat quality characteristics, and they will be included in our future experiments.

## 5. Conclusions

The findings of the current experiment strongly indicated that the marination of pork meat with yoghurt acid whey for 10–15 h improved tenderness without negative effects on the other quality traits, while no effect was observed after its immersion for 5 h. At the same time, the tenderness of chicken meat was not affected after YAW treatment for 10–15 h, but its oxidative stability was greatly improved. The addition of hesperidin did not have an additional or side effect on the quality traits of pork and chicken meat. The utilization of YAW in the meat industry can lead to the diminishment of hazards that are associated with its disposal, while an improvement in meat quality, tenderness, and oxidative stability in pork meat and oxidative stability in chicken meat is observed without further side effects on the other quality parameters.

## Data Availability

The data presented in this study are available on request from the corresponding author.
